# Histone H5 is a potent Antimicrobial Agent and a template for novel Antimicrobial Peptides

**DOI:** 10.1038/s41598-018-20912-1

**Published:** 2018-02-05

**Authors:** Joelle Jodoin, Maxwell T. Hincke

**Affiliations:** 1 Department of Cellular and Molecular Medicine, Faculty of Medicine, University of Ottawa, Ontario, K1H 8M5 Canada; 2 Department of Innovation in Medical Education, Faculty of Medicine, University of Ottawa, Ontario, K1H 8M5 Canada

## Abstract

Modern medicine is challenged continuously by the increasing prevalence of antibiotic resistant bacteria. Cationic antimicrobial peptides and their derivatives are interesting potential alternatives to antibiotics due to their rapid action, broad-spectrum of antimicrobial activity and limited emergence of bacterial resistance. This study reports the novel antimicrobial properties of histone H5, purified from chicken erythrocytes, and histone H5-derived synthetic peptides. Broth microdilution assays revealed that histone H5 has potent broad-spectrum antimicrobial activity against Gram-positive and Gram-negative planktonic bacteria (MIC range: 1.9 ± 1.8 to 4.9 ± 1.5 µg/mL), including vancomycin-resistant *Enterococcus* (VRE) and methicillin-resistant *Staphylococcus aureus* (MRSA). Moreover, histone H5 displayed anti-biofilm activity against established *Listeria monocytogenes* and *Pseudomonas aeruginosa* biofilms. Scanning electron microscopy demonstrated bacterial membrane damage after histone H5 treatment, while a hemolytic assay revealed that histone H5 is non-toxic towards mammalian erythrocytes, even at a concentration of 1 mg/mL. Although the predicted H5-derived antimicrobial peptides tested in this study were located within the antimicrobial domain of histone H5, their synthetic versions did not possess more potent antimicrobial activity than the full length protein. Overall, this study demonstrates that histone H5 is a potent antimicrobial and therefore a promising template for the development of novel histone H5-derived antimicrobial peptides.

## Introduction

The discovery of antibiotics has greatly changed global health by effectively treating bacterial infections. However, the emergence of otherwise antibiotic sensitive pathogens acquiring antibiotic resistance is becoming a worldwide health challenge. Causes of the antibiotic resistance crisis include the overuse and misuse of antibiotics; moreover, economic and regulatory obstacles have retarded the development of new antibiotics and slowed their approval^1,2^. Additionally, the extensive use of antibiotics in agriculture has a major impact on the spread of antimicrobial resistance. In fact, an estimated 80% of all antibiotics sold in the USA are used in livestock to control and treat bacterial infections as well as for growth promotion purposes^1,3^. Antibiotics used in animals bred for human consumption can lead to the development of antibiotic-resistant foodborne pathogens followed by their transmission to humans as food contaminants^4^. In addition to the increased risk of acquired illness from foodborne pathogens, efficient treatment of infections significantly decreases with the heightened development of antibiotic resistance within bacterial populations. In fact, it is estimated that approximately 700,000 deaths per year worldwide can be attributed to antimicrobial resistance, and that this death toll will increase to 10 million per year by 2050^5^.

The survival capabilities of bacteria are enhanced by the formation of biofilms on a variety of biotic and abiotic surfaces. Bacterial biofilms are a collection of unicellular organisms attached to a solid surface and enclosed in an extracellular polymeric substance (EPS) matrix^6,7^. Compared to freely suspended planktonic bacteria, biofilms are 10 to 1,000 times more resistant to antibiotics^7,8^. As a result of this enhanced resistance, biofilm-forming pathogens such as *Escherichia coli*, *Staphylococcus aureus* and *Pseudomonas aeruginosa*, have the capacity to colonize and infect medical devices (prosthetic joints, pacemakers, etc.); moreover, biofilms are of great concern in a variety of human diseases such as listeriosis (*Listeria monocytogenes*), endocarditis (*Enterococcus faecalis*) and cystic fibrosis (*P. aeruginosa*)^8,9^.

An antibiotic is generally defined to be a substance produced by one microorganism that selectively inhibits the growth of another. In contrast, antimicrobial peptides (AMPs) are widely distributed among a diverse range of organisms (prokaryotes, vertebrates, invertebrates and plants) and are an important component of the innate immune defence system^10^. Amongst these, CAMPs (cationic antimicrobial peptides) are short in length (12–50 amino acids), have an overall positive charge (net charge from +2 to +9) and contain a substantial proportion of hydrophobic residues (≥30%)^11–15^. CAMPs possess bactericidal activity that is based on the formation of electrostatic interactions with the anionic surfaces of bacterial membranes; such properties cannot be easily modified by most planktonic pathogens^16,17^. Consequently, development of resistance to CAMPs by bacteria is limited compared to that observed against antibiotics, although such antimicrobial peptides have been acting upon bacteria for hundreds of millions of years^18,19^. Therefore, the use of natural CAMPs as therapeutic agents has been widely investigated in recent years; however, high manufacturing costs have been a major barrier to their widespread use^20^. To address this issue, the identification of shorter peptides based upon the hydrophobicity, charge and amino acid composition of natural CAMPs is an effective strategy^20,21^. Accordingly, due to their broad-spectrum of antimicrobial activity and membrane-dependent mechanism of action, CAMPs and CAMP-derivatives are interesting candidates to overcome the global threat of antibiotic resistance.

Histone proteins share all of the essential traits of CAMPs; they are hydrophobic, cationic and can form amphipathic alpha-helical structures^16^. Consequently, many reports have demonstrated antimicrobial activities of histones. Our previous studies have demonstrated that a histone mixture (H1, H2A, H2B, H3, H4 and H5) extracted and purified from chicken erythrocytes possesses antimicrobial activity against a variety of Gram-negative and Gram-positive planktonic bacteria^22^, as well as eradication activity against Gram-positive bacterial biofilms^23^. Furthermore, our latest study demonstrated for the first time that purified erythrocyte-specific linker histone H5 is a potent antimicrobial peptide^23^. Histone H5 from chicken erythrocytes has a hydrophobic ratio of 28%, a total net charge of +61 and is 190 amino acids in length. Our preliminary results showed that purified H5 was equally effective against planktonic methicillin-sensitive and methicillin-resistant *Staphylococcus aureus*, and in this possessed more potent activity than the histone mixture^23^.

In this study, we further investigated the antimicrobial activity of histone H5 against a range of Gram-positive and Gram-negative planktonic bacteria, as well as against established *Pseudomonas aeruginosa* and *Listeria monocytogenes* bacterial biofilms. Furthermore, after a thorough bioinformatics analysis, six H5 peptide sequences with potential antimicrobial activity were identified, synthesized and tested for antimicrobial activity through preliminary screening. The most promising histone H5-derived peptide was synthesized (>95% purity) with different counterions, and the antimicrobial activity as well as secondary structures were compared to the full length histone H5 protein. The overall aim of this study was to further characterize the antimicrobial properties of histone H5 and to identify and develop novel histone H5-derived antimicrobial peptides.

## Results

### Assessment of purified histone H5 by densitometry and proteomics analysis

Our previous study reported an effective protocol to purify histone H5 from chicken erythrocytes by perchloric acid extraction and TCA precipitation, followed by ion exchange chromatography using a step salt gradient^23^. During the course of the present study, a total of five independent histone H5 preparations were assessed (Table 1). The purity of these histone H5 lots were determined by densitometry after SDS-PAGE and by proteomics analysis, revealing an average histone H5 purity of 98.6% ± 0.9 and 96.9% ± 1.8, respectively.

**Table 1:** Densitometry and proteomics analysis of purified histone H5.

Histone H5	Densitometry	Proteomics (LC/MS/MS)	Yield (µg H5/ g RBCs)	Starting material (g RBCs)
Extraction #1	98.9%	96.0%	0.5	12.7
Extraction #2	98.4%	96.8%	8.5	35.2
Extraction #3	97.0%	95.9%	41.1	62.9
Extraction #4	99.2%	95.9%	18.2	121.2
Extraction #5	99.3%	100%	286.0	101.0

Summary of the purity assessments of the independent histone H5 lots produced for this study. The negligible contaminants were determined by proteomics to be histone H1 variants (H1.11 R, H1.11 L, H1.01, H1.03).

### Antimicrobial activity of histone H5 versus planktonic bacteria

**Figure 1:**
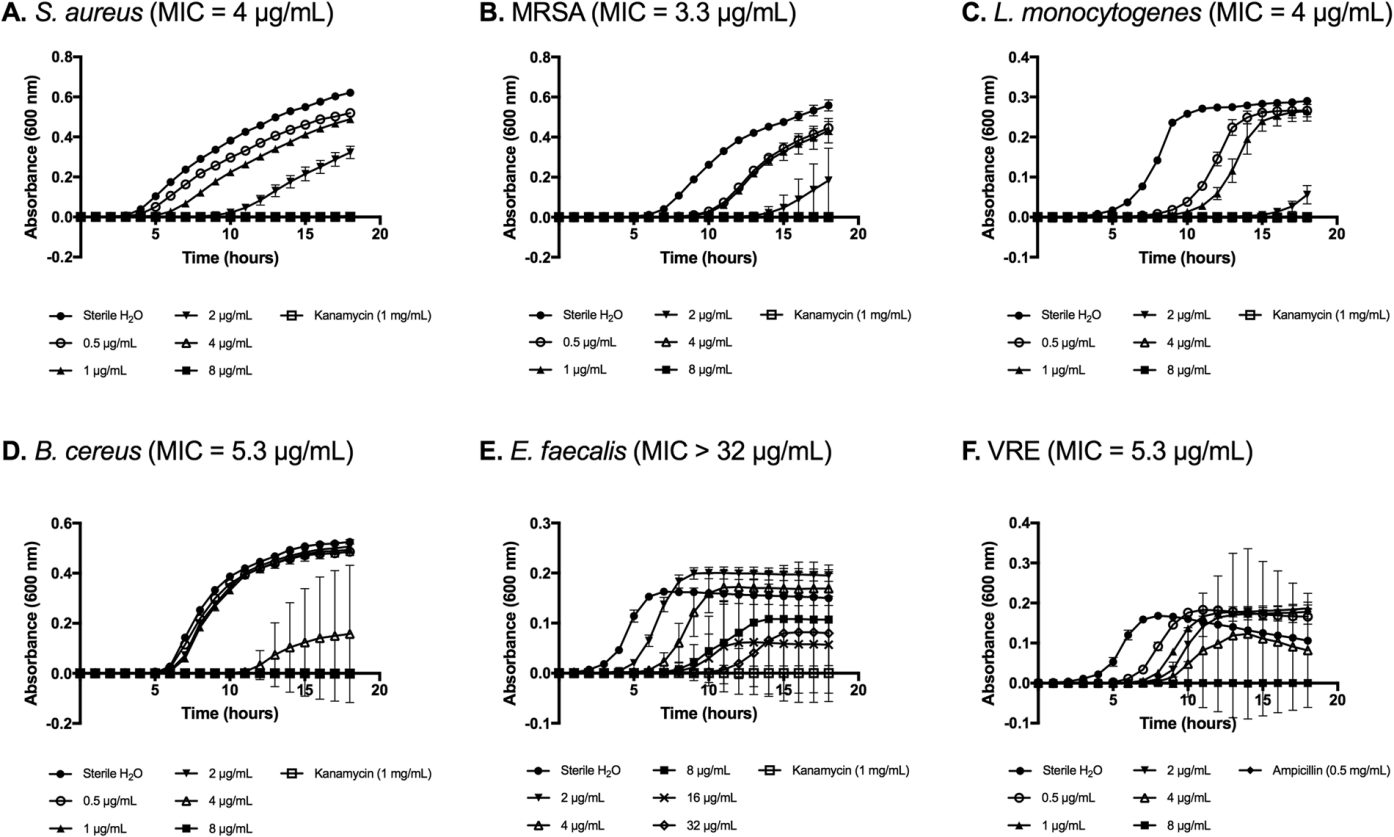
Dose-dependent growth inhibition of Gram-positive bacteria by histone H5. Minimum inhibitory concentrations (MICs) of histone H5 versus *S. aureus* (**A**), MRSA (**B**), *L. monocytogenes* (**C**), *B. cereus* (**D**), *E. faecalis* (**E**) and VRE (**F**) were determined by broth microdilution assays. Sterile ddH_2_O, pH 7.4, was the negative control for inhibition. Kanamycin (1 mg/mL) or ampicillin (0.5 mg/mL) was the positive control for inhibition. Results are representative of three independent trials, each performed in triplicate (n = 3).

**Figure 2:**
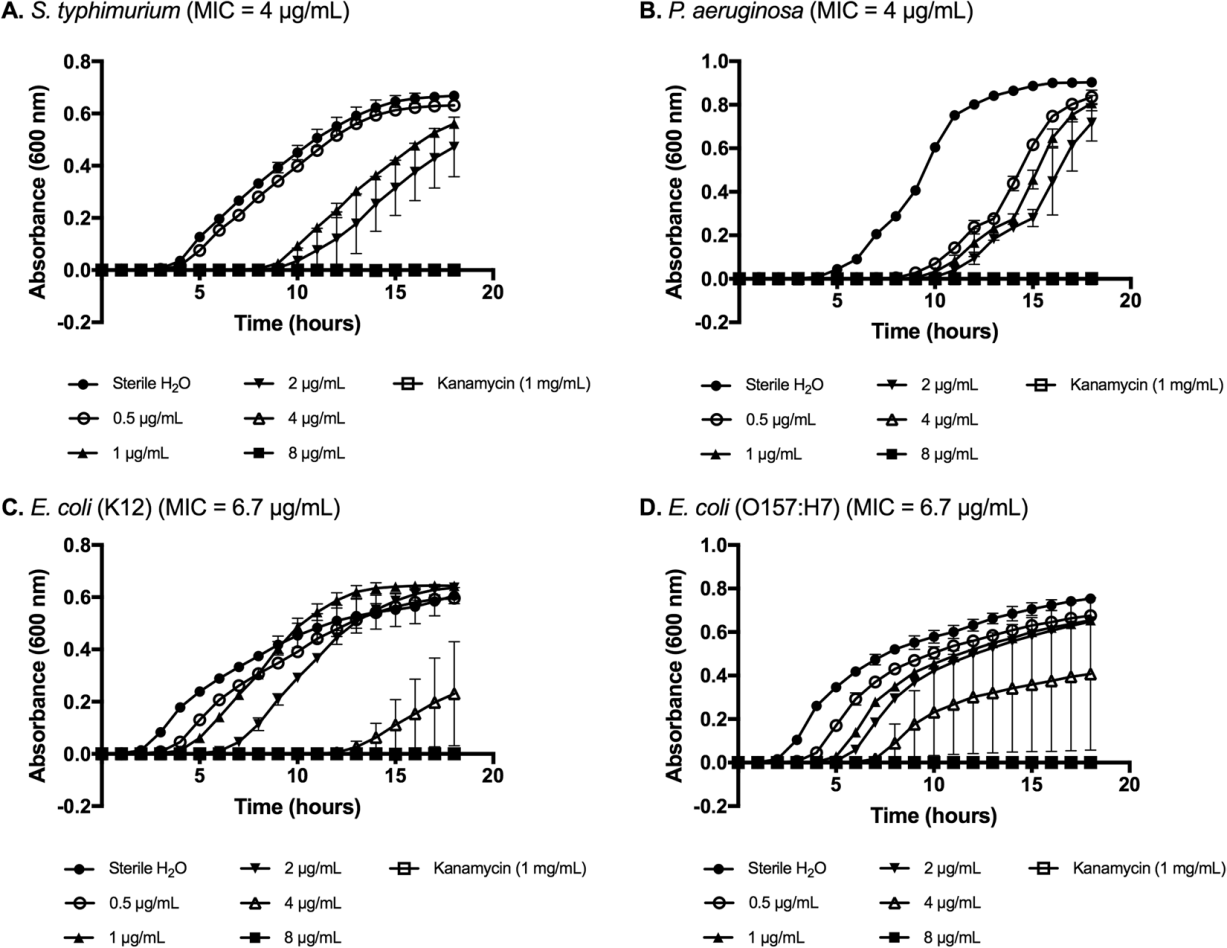
Dose-dependent growth inhibition of Gram-negative bacteria by histone H5. Minimum inhibitory concentrations (MICs) of histone H5 versus *S. typhimurium* (**A**), *P. aeruginosa* (**B**), *E. coli* (K12) (**C**) and *E. coli* (O157:H7) (**D**) were determined by broth microdilution assays. Sterile ddH2O, pH 7.4, was the negative control for inhibition. Kanamycin (1 mg/mL) was the positive control for inhibition. Results are representative of three independent trials, each performed in triplicate (n = 3).

Antimicrobial activity of histone H5 against a variety of Gram-positive and Gram-negative planktonic bacteria was evaluated using a broth microdilution assay. The average minimum inhibitory concentration (MIC) and minimum bactericidal concentration (MBC) values (mean ± SD, n = 3) for six Gram-positive and four Gram-negative bacterial species are shown in Table 2. As revealed in Figs 1 and 2, bacterial growth inhibition by histone H5 was dose-dependent. The MIC values for histone H5 against Gram-positive bacteria *B. cereus*, *E. faecalis*, *L. monocytogenes*, MRSA, *S. aureus* and VRE were 3.8 ± 1.7 µg/mL, >32 µg/mL, 4 ± 0 µg/mL, 2.4 ± 0.8 µg/mL, 3.8 ± 0.4 µg/mL and 4 ± 1.2 µg/mL, respectively. *E. faecalis* was the least susceptible to histone H5 inhibition, requiring over 32 µg/mL for complete bacterial growth inhibition which was the highest histone H5 concentration tested. The MIC values for all other Gram-positive pathogens were not significantly different from each other, demonstrating similar susceptibilities to histone H5, including antibiotic-resistant bacteria MRSA and VRE. The MIC values for histone H5 versus Gram-negative bacteria *E. coli* K12, *E. coli* O157:H7, *P. aeruginosa* and *S. typhimurium* were 4.9 ± 1.5 µg/mL, 4.9 ± 1.5 µg/mL, 2.9 ± 1 µg/mL and 1.9 ± 1.8 µg/mL, respectively. Thus, all of the Gram-negative bacterial pathogens tested for growth inhibition by histone H5 showed similar susceptibilities to the purified H5 protein.

**Table 2:** Summary of MIC and MBC values of histone H5 against planktonic bacteria.

Planktonic bacteria	MIC (µg/mL)	MBC (µg/mL)
**Gram-positive**		
*B. cereus*	3.8 ± 1.7	3.8 ± 1.7
*E. faecalis*	>32	>32
*L. monocytogenes*	4 ± 0	4.4 ± 0.8
MRSA	2.4 ± 0.8	2.7 ± 1.2
*S. aureus*	3.8 ± 0.4	4 ± 0
VRE	4 ± 1.2	4.4 ± 1.9
**Gram-negative**		
*E. coli* (K12)	4.9 ± 1.5	6.7 ± 2.3
*E. coli* (O157:H7)	4.9 ± 1.5	4.9 ± 1.5
*P. aeruginosa*	2.9 ± 1	2.9 ± 1
*S. typhimurium*	1.9 ± 1.8	1.9 ± 1.8

Minimum inhibitory concentration (MIC) values (mean ± SD, n = 3) and minimum bactericidal concentration (MBC) values (mean ± SD, n = 3) of purified chicken erythrocyte histone H5 versus Gram-positive and Gram-negative planktonic bacteria were determined by broth microdilution assays.

### Scanning electron microscopy (SEM)

**Figure 3:**
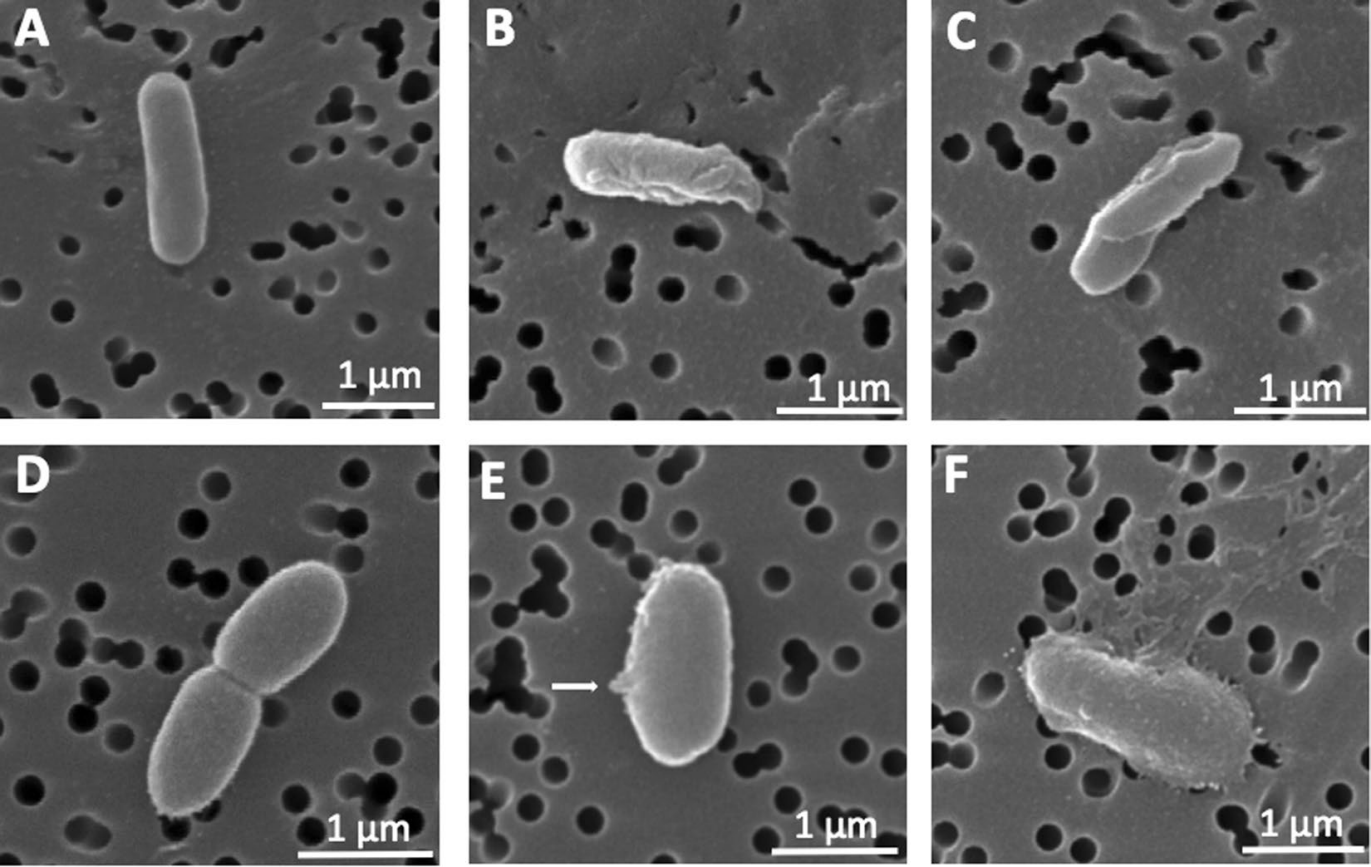
Scanning electron microscopy (SEM) of histone H5-treated *Listeria monocytogenes* and *Pseudomonas aeruginosa* planktonic cells. (**A**) *L. monocytogenes* treated with ddH2O (untreated control cells); (**B**) and (**C**) *L. monocytogenes* treated with 4 µg/mL of histone H5; (**D**) *P. aeruginosa* treated with ddH2O (untreated control cells); (**E**) and (**F**) *P. aeruginosa* treated with 4 µg/mL of histone H5. Pore formation is indicated by the white arrow (**E**). Results are representative of three independent trials (n = 3). All at 30,000X magnification.

In order to study the effect of histone H5 on bacterial membranes, Gram-positive and Gram-negative planktonic bacteria incubated with histone H5 were visualized by scanning electron microscopy (SEM). Representative SEM images of *Listeria monocytogenes* and *Pseudomonas aeruginosa* untreated (H_2_O control) and histone H5-treated bacterial cells are shown in Fig. 3. Untreated *L. monocytogenes* (Fig. 3A) and *P. aeruginosa* (Fig. 3D) bacteria showed normal and smooth surface structures, whereas clear morphological differences were observed after the histone H5 treatment in both bacterial species. Multiple indentations in the bacterial membrane, membrane wrinkling and pronounced deformations were visualized in the *L. monocytogenes* cells after exposure to 4 µg/mL of histone H5 (MIC value = 4 ± 0 µg/mL) (Fig. 3B,C). Similarly, *P. aeruginosa* bacterial cells subjected to 4 µg/mL of histone H5 treatment (MIC value = 2.9 ± 1 µg/mL) showed indentations in the bacterial membrane, small pore formation (Fig. 3E), and fibrous material possibly originating from bacterial content leakage (Fig. 3F). Similar observations were made in each of three independent trials, each performed in duplicate.

### MBEC assay to evaluate histone H5 anti-biofilm activity

**Figure 4:**
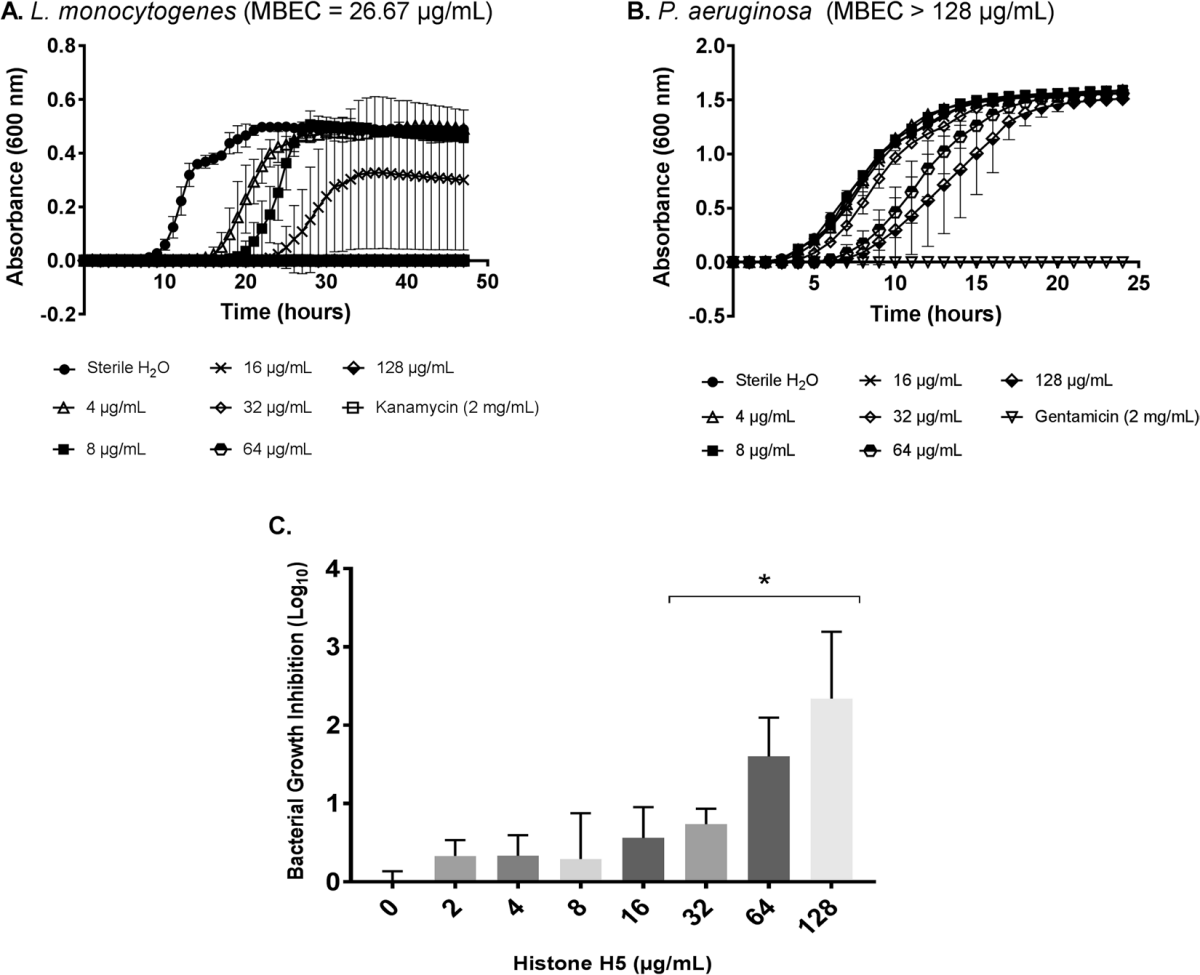
Dose-dependent growth inhibition of Gram-positive and Gram-negative bacterial biofilms by histone H5. Minimum biofilm eradication concentrations (MBECs) of purified histone H5 versus *L. monocytogenes* (**A**) and *P. aeruginosa* (**B**). Sterile ddH_2_O, pH 7.4, was the negative control for inhibition. Kanamycin (2 mg/mL) or gentamicin (2 mg/mL) was the positive control for inhibition. Results are representative of three independent trials, each performed in triplicate (n = 3). (**C**) Dose-dependent logarithmic growth inhibition of *P. aeruginosa* bacterial biofilms by histone H5. Results are a summary of three independent trials, each performed in triplicate (n = 3). Statistical analysis was done by Student’s T-Test; (*) indicates P ≤ 0.04 compared with the control (0 µg/mL of histone H5).

The anti-biofilm activity of histone H5 against *P. aeruginosa* and *L. monocytogenes* biofilms was assessed using the MBEC Assay Biofilm Inoculator (formerly the Calgary Biofilm Device (CBD)). Between 10^6^–10^7^ CFUs/peg was obtained for uninhibited *P. aeruginosa* biofilms, while 10^5^ CFUs/peg was obtained for uninhibited *L. monocytogenes* biofilms. The average minimum biofilm eradication concentration (MBEC) values (mean ± SD, n = 3) for the two bacterial species tested, are shown in Table 3. The MBEC value for the purified H5 protein against *L. monocytogenes* was 19.1 ± 13.1 µg/mL, while *P. aeruginosa* had an MBEC value > 128 µg/mL. Although histone H5 was unable to completely eradicate *P. aeruginosa* biofilms at a concentration of ≤128 µg/mL, a dose-dependent response to the increasing concentrations of histone H5 was observed (Fig. 4B,C). Significant growth inhibition was detected at 32 µg/mL (0.7 ± 0.2 log inhibition), 64 µg/mL (1.6 ± 0.5 log inhibition) and 128 µg/mL (2.3 ± 0.9 log inhibition) of histone H5 treatment compared to the untreated control cells (Fig. 4C, P value < 0.04).

**Table 3:** Summary of MBEC values of histone H5 against bacterial biofilms.

Bacterial biofilms	MBEC (µg/mL)
**Gram-positive**	
*L. monocytogenes*	19.1 ± 13.1
**Gram-negative**	
*P. aeruginosa*	>128

Minimum biofilm eradication concentration (MBEC) values (mean ± SD, n = 3) were determined for purified histone H5 versus Gram-positive and Gram-negative bacterial biofilms.

### Hemolytic assessment of histone H5 against mammalian erythrocytes

The potential for histone H5 toxicity towards mammalian cells was assessed using a hemolytic assay. A wide range of histone H5 concentrations against rat erythrocytes were tested (from 0.005 to 1 mg/mL) (Table 4). Statistical analysis revealed that hemolysis after treatment with 1, 0.5, 0.025, 0.0125 and 0.005 mg/mL of histone H5 was not significantly different than the negative control (PBS). Therefore, even at the highest concentration tested (1 mg/mL), histone H5 is non-hemolytic.

**Table 4:** Hemolytic activity of histone H5 versus mammalian RBCs.

Concentration of histone H5 (mg/mL)	Hemolysis %^a^
1	2.0 ± 1.4^b^
0.75	2.8 ± 1.3
0.5	2.4 ± 1.4^b^
0.25	2.6 ± 0.6
0.125	3.2 ± 1.1
0.05	2.3 ± 0.6
0.025	1.8 ± 1.6^b^
0.0125	0.5 ± 0.5^b^
0.005	0.1 ± 0.4^b^

A wide range of histone H5 concentrations (up to 1 mg/mL) were tested against rat erythrocytes. Controls include PBS and 0.05% Triton-X, representing no hemolysis and 100% hemolysis, respectively. Hemolysis % values represent the mean ± SD of three independent trials, each performed in triplicate (n = 3).

a. Hemolysis % was calculated according to the following equation: hemolysis (%) = ((OD540 nm sample −OD540 nm no hemolysis)/(OD540 nm 100% hemolysis −OD540 nm no hemolysis)) × 100.

b. No significant hemolytic activity of histone H5 was observed, compared to the PBS (no hemolysis) control.

### Characterization of antimicrobial properties of histone H5-derived peptides

#### Bioinformatics analysis

**Figure 5:**
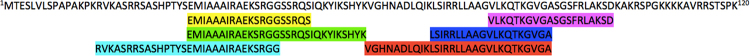
Alignment of histone H5 with six histone H5-derived peptides possessing predicted antimicrobial activity. Alignment of the peptide sequences with the first 120 amino acid residues of the full-length histone H5 protein (full length histone H5: 190 amino acids). All predicted active sequences are within amino acid positions 15 to 100, potentially representing the active antimicrobial domain(s) of histone H5.

A thorough bioinformatics analysis was completed based upon consideration of the general characteristics of known antimicrobial peptides, as implemented in the Antimicrobial Peptide Database (APD: http://aps.unmc.edu/AP/main.php). Six histone H5-derived peptide sequences with potential antimicrobial activity were identified (Table 5 and Fig. 5). These peptides have a net charge ranging from +2 to +5, contain a substantial proportion of hydrophobic residues (30% to 50%), have at least 3 hydrophobic residues on the same surface of the alpha-helical structure and have a maximum similarity of 46% with any other known AMP in the database.

**Table 5:** Characteristics of the six histone H5-derived peptides. An *in silico* analysis identified six histone H5 peptide sequences with potential antimicrobial activity. Properties such as length of the peptide and where it is located within the original H5 sequence, charge, hydrophobicity, number of hydrophobic residues on the same surface and highest similar AMP are shown in the table. All information was retrieved from the Antimicrobial Peptide Database (APD: http://aps.unmc.edu/AP/main.php).

Sequence	# of a.a.^1^	Charge	Hydrophobicity	# of hydrophobic residues on the same surface^2^	Highest similarity with AMP in database^3^
EMIAAAIRAEKSRGGSSRQS	20 (31 to 50)	+2	35%	5	35% (Pantinin-2)
EMIAAAIRAEKSRGGSSRQSIQKYIKSHYK	30 (31 to 60)	+5	30%	7	33.33% (Rugosin)
RVKASRRSASHPTYSEMIAAAIRAEKSRGG	30 (15 to 45)	+5	33%	5	35.13% (Cecropin P3)
VLKQTKGVGASGSFRLAKSD	20 (81 to 100)	+3	35%	3	40% (Caerin 4.2)
LSIRRLLAAGVLKQTKGVGA	20 (71 to 90)	+4	50%	5	46.15% (Caerin 2.1)
VGHNADLQIKLSIRRLLAAGVLKQTKGVGA	30 (61 to 90)	+4	46%	5	39.39% (Caerin 2.1)

^1^Amino acid sequence length (where it is located in original H5 sequence).

^2^Each peptide is predicted to form an alpha-helix. The website algorithm can also predict how many hydrophobic residues are on the same surface. If ≤2 hydrophobic residues on the same surface, it is predicted that the peptide will not be an AMP.

^3^The website also allows us to do an alignment to find the 5 most similar peptides in the database (>2900 AMPs in the database). The highest similar AMP in the database for each peptide is shown in the column.

#### Preliminary peptide screening for antimicrobial activity against planktonic bacteria

Approximately 5 mg of lyophilized powder (purities ranging from 50% to 81.9%) for each histone H5-derived peptide was synthesized. The estimated purity of each peptide is as follows: H5(16–45) 57.7%, H5(61–90) 81.4%, H5(71–90) 80.9%, H5(81–100) 78.1%, H5(31–50) 50% and H5(31–60) 81.9%. The quantity of each peptide was sufficient to complete a preliminary peptide screen to test their activity against one Gram-positive (*Listeria monocytogenes*) and one Gram-negative (*Pseudomonas aeruginosa*) planktonic bacteria in three independent trials, each in triplicate (up to 64 µg/mL). The average MIC values (mean ± SD, n = 3) are shown in Table 6. Only histone H5-derived peptide H5(61–90) displayed complete bacterial growth inhibition within the concentration range tested versus *Listeria monocytogenes*, possessing a MIC value of 23.1 ± 8.1 µg/mL. The peptide H5(71–90) was unable to completely inhibit *L*. *monocytogenes* bacterial growth, but displayed significant growth inhibition at 64 µg/mL (4.1 ± 1.0 log inhibition, P value ≤ 0.02) compared to the untreated control cells (0 µg/mL), while the other four peptides (H5(16–45), H5(81–100), H5(31–50) and H5(31–60)) were inactive at the highest concentration of 64 µg/mL. The H5-derived peptides were also tested against *Pseudomonas aeruginosa*, with MIC values of 16 ± 0 µg/mL (H5(61–90)), 26.7 ± 14.1 µg/mL (H5(71–90)) and non-inhibitory at the highest concentration of 64 µg/mL (H5(16–45), H5(81–100), H5(31–50) and H5(31–60)). The histone H5-derived peptide H5(61–90) is thus the only peptide which displayed complete bacterial growth inhibition against *L. monocytogenes* within the tested concentration range (1 µg/mL – 64 µg/mL) and possessed the most potent antimicrobial activity versus *P. aeruginosa* compared to the other peptides.

**Table 6:** Summary of MIC values of histone H5-derived peptides against planktonic bacteria.

Planktonic bacteria	Peptide H5 (16–45)	Peptide H5 (61–90)	Peptide H5 (71–90)	Peptide H5 (81–100)	Peptide H5 (31–50)	Peptide H5 (31–60)
**Gram-positive**						
*Listeria monocytogenes*	**NI** ^**‡**^	**23.1** ± **8.1**	>**64**^†^	**NI** ^**‡**^	**NI** ^**‡**^	**NI** ^**‡**^
**Gram-negative**						
*Pseudomonas aeruginosa*	**NI** ^**‡**^	**16** **±** **0**	**26.7**±**14.1**	**NI** ^**‡**^	**NI** ^**‡**^	**NI** ^**‡**^

Minimum inhibitory concentration (MIC) values (mean ± SD, n = 3) of the histone H5-derived peptides versus Gram-positive (*L. monocytogenes*) and Gram-negative (*P. aeruginosa*) planktonic bacteria.

^†^Significant bacterial growth inhibition was detected at 64 µg/mL (4.1 ± 1.0 log inhibition, P value ≤ 0.02) compared to the untreated control cells (0 µg/mL).

^‡^Non-inhibitory at the highest concentration tested (64 µg/mL).

### Antimicrobial properties of purified (>95%) histone H5-derived peptide H5(61–90)

**Figure 6:**
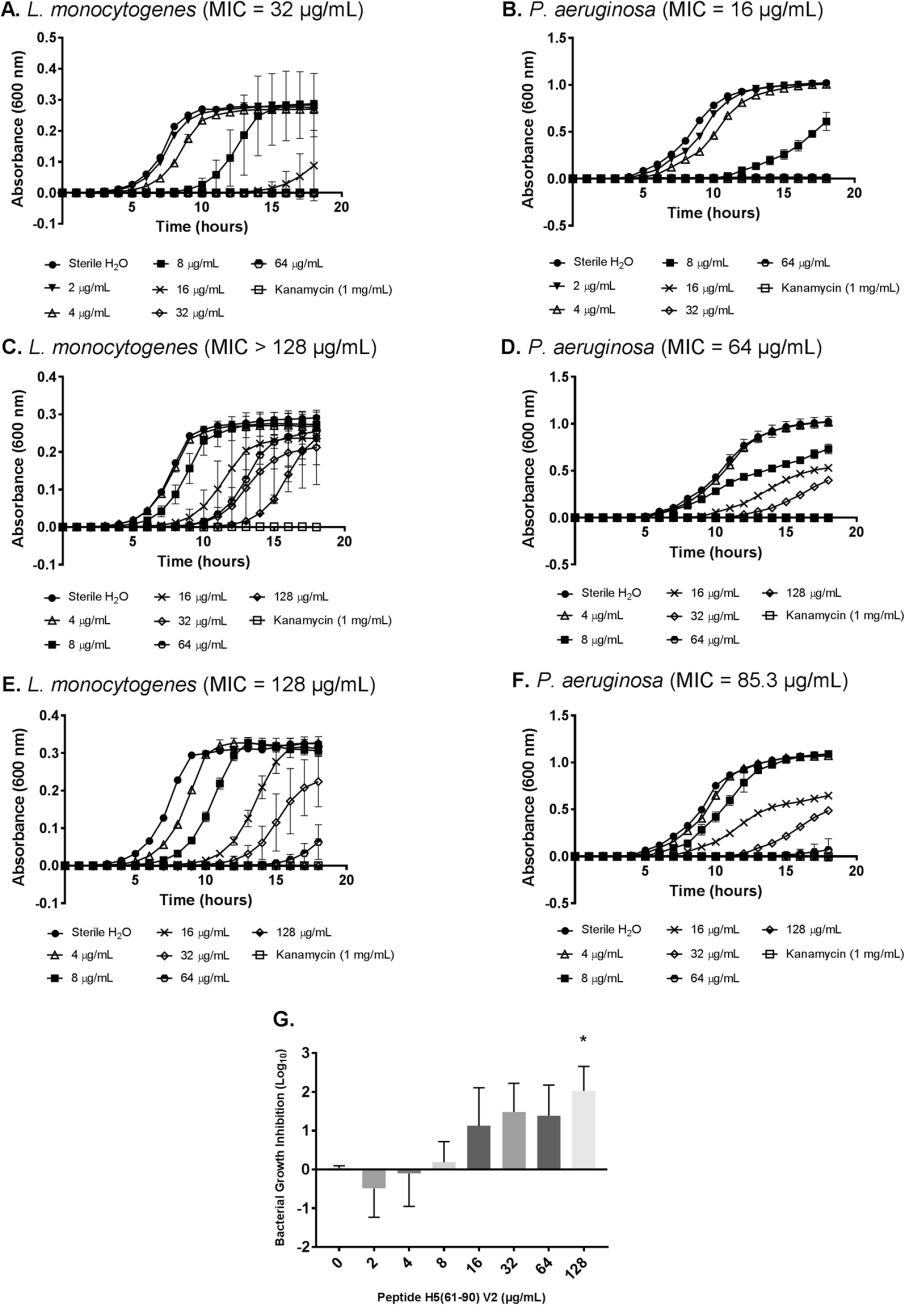
Dose-dependent growth inhibition of Gram-positive and Gram-negative planktonic bacteria by histone H5-derived peptides H5(61–90) V1-V3. MICs of peptide H5(61–90) V1 versus *L. monocytogenes* (**A**) and *P. aeruginosa* (**B**), of peptide H5(61–90) V2 versus *L. monocytogenes* (**C**) and *P. aeruginosa* (**D**) and of peptide H5(61–90) V3 versus *L. monocytogenes* (**E**) and *P. aeruginosa* (**F**) determined by broth microdilution assays. Sterile ddH2O, pH 7.4, was the negative control for inhibition. Kanamycin (1 mg/mL) was the positive control for inhibition. Results are representative of three independent trials, each performed in triplicate (n = 3). (**G**) Dose-dependent logarithmic growth inhibition of *Listeria monocytogenes* planktonic bacteria by peptide H5(61–90) V2. Results are a summary of three independent trials, each performed in triplicate (n = 3). Statistical analysis was done by Student’s T-Test, (*) indicates P ≤ 0.03 compared with the control (0 μg/mL of peptide H5(61–90) V2).

Preliminary peptide screening of the six histone H5-derived peptides with potential antimicrobial activity revealed that peptide H5(61–90) had the most potent antimicrobial activity compared to the other peptides. Accordingly, two additional lots of peptide H5(61–90) were synthesized at a purity exceeding 95%: with either guaranteed TFA removal (replaced with hydrochloric salt) (H5(61–90) V2), or without TFA removal (H5(61–90) V3). Both versions were evaluated since it has been reported that TFA can affect the secondary structure of peptides. For example, TFA modified the structural folding of pediocin, a 44-amino acid bacteriocin, whereas HCl did not affect its conformation^24^. The antimicrobial activity of these peptides against Gram-positive and Gram-negative planktonic bacteria was evaluated using broth microdilution assays. The average MIC values (mean ± SD, n = 3) are shown in Table 7. The MIC values for histone H5-derived peptide H5(61–90) V2 against *L. monocytogenes* and *P. aeruginosa* were>128 µg/mL and 33.8 ± 26.3 µg/mL, respectively. The MIC values for histone H5-derived peptide H5(61–90) V3 against *L. monocytogenes* and *P. aeruginosa* were 106.7 ± 37.0 µg/mL and 48.0 ± 32.4 µg/mL, respectively. *L. monocytogenes* was less susceptible to the histone H5-derived peptide H5(61–90) V2, requiring over 128 µg/mL which was the highest concentration tested. Although H5(61–90) V2 was unable to completely inhibit *L. monocytogenes* bacterial growth, a dose-dependent response to the increasing concentrations of the peptide was observed (Fig. 6C and Fig. 6G). Furthermore, a significant growth inhibition was detected at 128 µg/mL (2.0 ± 0.6 log inhibition) of H5(61–90) V2 treatment compared to the untreated control cells (0 µg/mL) (Fig. 6G, P value ≤ 0.03). There is no significant difference between the MIC values of all three versions of the peptide versus *P. aeruginosa*, which demonstrates similar susceptibilities to the peptides (Table 7 and Fig. 6).

**Table 7:** Summary of the characteristics and MIC values of histone H5-derived peptides H5(61–90).

Histone H5-derived peptide	Purity (%)	Salt Form	Antimicrobial activity against *L. monocytogenes* (*µg/mL*)	Antimicrobial activity against *P. aeruginosa (µg/mL)*
H5(61–90) V1	81.4	TFA	23.1 ± 8.1	16 ± 0
H5(61–90) V2	96.5	HCl	>128	33.8 ± 26.3
H5(61–90) V3	95.5	TFA	106.7 ± 37.0	48.0 ± 32.4

The purities, salt forms and antimicrobial properties of the three versions of peptide H5(61–90) are listed in this table. TFA = trifluoroacetic acid; HCl = hydrochloric acid. Minimum inhibitory concentration (MIC) values (mean ± SD, n = 3) of the peptides H5(61–90) versus Gram-positive (*L. monocytogenes*) and Gram-negative (*P. aeruginosa*) planktonic bacteria.

### Circular dichroism (CD) analysis of histone H5 and histone H5-derived peptides H5(61–90)

**Figure 7:**
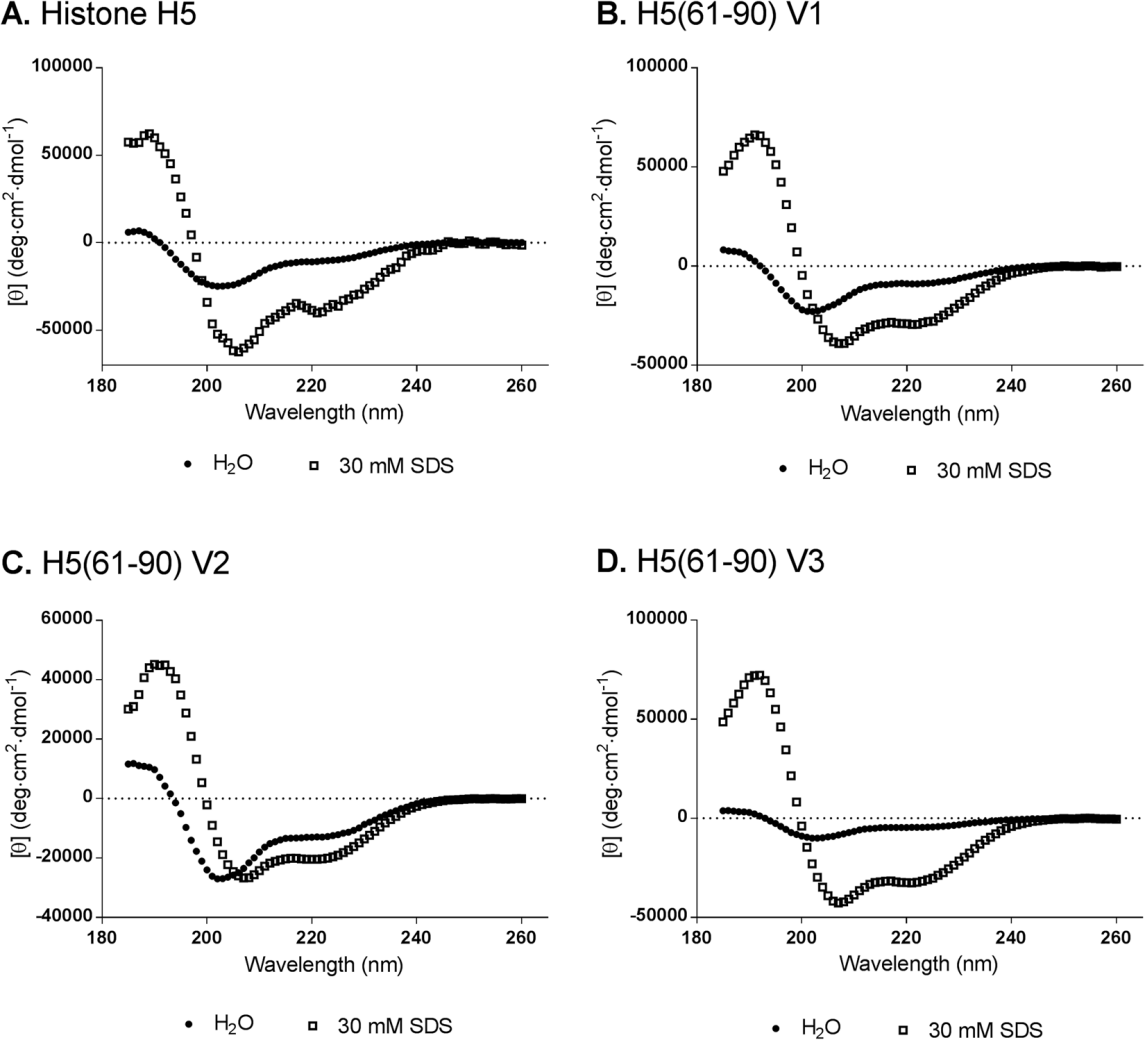
CD spectra of histone H5 and histone H5-derived peptides. CD spectra of histone H5 and histone H5-derived peptides dissolved in sterile ddH2O pH 7.4 (●) or 30 mM SDS (□), recorded at room temperature.

The secondary structure of histone H5 and the three preparations of histone H5-derived peptide H5(61–90) was investigated in different environments: sterile H_2_O, pH 7.4 (aqueous environment) and 30 mM SDS (mimicking the negatively charged bacterial membrane environment), by circular dichroism (CD) spectroscopy. The superimposition of the CD spectrum in different environments, for each peptide, is shown in Fig. 7. Histone H5 and the histone H5-derived peptides displayed random coil secondary structures in the aqueous environment; however, in the presence of 30 mM SDS, the spectra of the peptides displayed an α-helical conformation (evidenced by the minima at approximately 222 nm and 208 nm as well as the positive band at 193 nm). Deconvolution of the spectral data with the CDSSTR program estimated the secondary structural components (α-helix, β-sheet, turns, etc.) of each peptide (Table 8). The calculated α-helical component of full length histone H5 was 23.4%, while the histone H5-derived peptides had α-helical content of 47.7% (H5(61–90) V1), 41.5% (H5(61–90) V2) and 6.1%(H5(61–90) V3) in the aqueous environment. However, the α-helical proportions in the presence of 30 mM SDS were 76.9%, 84.2%, 75.5% and 84.7% for histone H5, H5(61–90) V1, H5(61–90) V2 and H5(61–90) V3, respectively.

**Table 8:** Predicted percentages of secondary structure components of histone H5 and of histone H5-derived peptides.

	H_2_O (%)				30 mM SDS (%)			
	Histone H5	H5(61–90) V1	H5(61–90) V2	H5(61–90) V3	Histone H5	H5(61–90) V1	H5(61–90) V2	H5(61–90) V3
α-helix	23.4	47.7	41.5	6.1	76.9	84.2	75.5	84.7
β-sheet	22.7	18.7	15.0	34.7	9.7	4.1	3.2	4.2
Turns	22.4	17.4	18.2	24.0	4.5	2.7	5.9	2.1
Random Coil	32.2	16.6	25.5	34.2	8.9	8.9	14.2	8.3

Deconvolution of circular dichroism data for each peptide sample was accomplished using the CDSSTR software^56^.

## Discussion

The overuse and misuse of antibiotics in agriculture and medicine has led to the emergence of antibiotic-resistant bacterial pathogens. Therefore, the development of novel alternatives to treat infections is an urgent necessity. Cationic antimicrobial peptides (CAMPs) are intriguing candidates as anti-infective therapeutic compounds since they show rapid bactericidal activity against a broad spectrum of microorganisms, and have been observed to induce limited bacterial resistance due to their main target being the bacterial membranes^20,25^. Histones are generally known as proteins that package and regulate the transcription of DNA. However, they also possess all of the essential characteristics of CAMPs necessary for antimicrobial activity^16^. Histone H5, an archetypal CAMP, is a nucleated-erythrocyte-specific histone with facile extraction and purification from chicken blood, which is a major poultry industry waste product. Our previous study reported that histone H5 is a potent antimicrobial peptide against methicillin-sensitive and methicillin-resistant *S. aureus*^23^. This study documents additional and novel antimicrobial properties of histone H5 and histone H5-derived peptides.

Once extracted and purified (>97%) from chicken erythrocytes, the antimicrobial activity of histone H5 was tested against six Gram-positive and four Gram-negative planktonic bacterial strains. The bacteria tested in this study showed similar susceptibilities to the protein (MIC range: 1.9 ± 1.8 to 4.9 ± 1.5 µg/mL), except for *E. faecalis* which was significantly less susceptible to complete growth inhibition, requiring >32 µg/mL (Table 2). Human β-defensin-3, a human host defence antimicrobial peptide, has a comparable spectrum of antimicrobial activity, with MIC values of 6, 12, 13 and >512 µg/mL against *E. coli*, *S. aureus*, *P. aeruginosa* and *E. faecalis*, respectively^26,27^. Several CAMPs, such as indolicidin, ranalexin, magainin II and LL-37, have a broad-spectrum of antimicrobial activity against microorganisms, including Gram-positive and Gram-negative bacteria^28,29^. Similarly, histone H5 demonstrated potent broad-spectrum antimicrobial activity.

Bactericidal activity, the capability of an agent to kill bacteria, can be recognized when the MBC value is ≤4 times the MIC value^30^. In this study, the MIC and MBC values for each bacterial strain (Gram-positive and Gram-negative) were not significantly different from each other (Table 2; P ≤ 0.05) indicating that the antimicrobial activity of histone H5 is bactericidal in contrast to bacteriostatic. Thus, histone H5 exerts potent broad-spectrum antimicrobial activity that ultimately leads to bacterial cell death (MBC/MIC ≤ 4). Other histones have also been shown to possess bactericidal activity, including histones extracted from calf thymus, and histones H1 and H2B extracted and purified from the chicken reproductive system^31,32^. However, our work is the first reported assessment of the antimicrobial activity of purified histone H5.

The majority of CAMPs display bactericidal activity through the establishment of electrostatic interactions with bacterial membranes, followed by membrane permeabilization and pore formation. Previous research from our laboratory has demonstrated that a histone mixture extracted from chicken erythrocytes has the ability to bind to lipopolysaccharides (LPS) and lipoteichoic acids (LTA), components of the cell wall in Gram-negative and Gram-positive bacteria, respectively^22^. In this study, the effect of histone H5 on bacteria surfaces was investigated through scanning electron microscopy (SEM). *Listeria monocytogenes*, a major foodborne pathogen, and *Pseudomonas aeruginosa*, an important nosocomial and resistant pathogen, were treated with 4 µg/mL of histone H5, followed by assessment of bacterial cell morphology by SEM. After histone H5 treatment, damage to the bacterial cell surfaces, indicated by loss of cell smoothness, pronounced deformations, pore formation and leakage of cytoplasmic content was observed (Fig. 3). Similarly, other CAMPs including RI18, a PMAP-36-derived peptide, and Sphistin, a crab histone H2A-derived peptide, also induced bacterial cell surface damage when tested against *Escherichia coli* and *Staphylococcus aureus*, respectively^21,33^. On the contrary, other histone-derived antimicrobial peptides, such as buforin II, DesHDAP1 and DesHDAP2, can induce bacterial cell death by translocating through the lipid bilayer without causing significant membrane permeabilization^34–36^. The observed effect reported for histone H5 on bacterial cell surfaces, however, is indicative of a membrane dependent mechanism of bactericidal activity.

*L. monocytogenes* and *P. aeruginosa* are major human pathogens that cause serious illnesses through foodborne and hospital-acquired infections, respectively. In fact, from 1995 to 2004, severe infections by *L. monocytogenes* resulted in 43 deaths in Canada, and an estimated 260 deaths in the United States from 2000 to 2008^37,38^. Moreover, an estimated 51,000 healthcare-associated infections leading to 440 deaths per year in the United States are caused by *P. aeruginosa*^39^. Additionally, both pathogens have the ability to form biofilms, which enhances their persistence in unfavourable environments and further decreases the effectiveness of antibiotics^8^. In this study, the biofilm eradicating capabilities of histone H5 were tested against *L. monocytogenes* and *P. aeruginosa* biofilms. Histone H5 was unable to completely eradicate *P. aeruginosa* biofilms within the concentrations tested and, therefore, has an unknown MBEC value > 128 µg/mL (Table 3). Other CAMPs, including LL-37, CAMA, melittin, defensin and magainin II, have been tested against *P. aeruginosa* biofilms, with MBEC values of 640 µg/mL for LL-37 and CAMA (MBEC/MIC ratio of 5–80), and unknown MBEC values > 640 µg/mL for melittin, defensin and magainin II^40^. Although complete *P. aeruginosa* biofilm eradication was not obtained in our study, histone H5 induced a dose-dependent response to its increasing concentrations which significantly inhibited bacterial growth at 32, 64 and 128 µg/mL (Fig. 4C). Considering the MIC value of 2.9 ± 1 µg/mL against planktonic *P. aeruginosa* and the significant biofilm growth inhibition by H5 observed at concentrations as low as 32 µg/mL, we would expect complete *P. aeruginosa* biofilm eradication at elevated histone H5 concentrations, as seen with LL-37 and CAMA. This hypothesis would have to be tested in future studies that utilize higher levels of H5. On the other hand, histone H5 was able to eradicate *L. monocytogenes* biofilms at a concentration of 19.1 ± 13.1 µg/mL, which gives an approximate MBEC/MIC ratio of 5 fold (Table 3). Paenibacterin, an antimicrobial lipopeptide produced by *Paenibacillus thiaminolyticus*, has been tested against established *L. monocytogenes* biofilms. After 2 and 4 hours of treatment, paenibacterin (6.8 µg/mL) was effective in disrupting established *L. monocytogenes* biofilms developed statically at 30 °C, but not effective in destroying stronger biofilms developed at 37 °C^41^. While several CAMPs have been previously shown to inhibit *L. monocytogenes* biofilm formation, histone H5 and paenibacterin are the first antimicrobial peptides to our knowledge to possess complete *L. monocytogenes* biofilm eradication activity under the experimental conditions tested. Accordingly, histone H5 has the potential to be developed as an effective anti-biofilm agent aimed at minimizing *L. monocytogenes* persistence in the food industry and therefore preventing new outbreaks involving this major foodborne pathogen.

CAMPs have a non-specific mode of action and mainly target the bacterial cell wall, making the development of bacterial resistance difficult. Unfortunately, this characteristic can also cause plasma membrane damage in mammalian cells, making many antimicrobial peptides unsuitable for clinical applications. As amphipathic structures, CAMPs have exposed hydrophobic domains that can potentially interact with RBC membrane protein constituents, cholesterol or phospholipids, leading to hemolysis^42^. Gramicidin S, piscidin I and dermaseptin S4 are examples of CAMPs that display strong hemolytic activity towards mammalian red blood cells (RBCs), which unfortunately restricts clinical use of Gramicidin S to topical applications^43,44^. In order to evaluate the ability of histone H5 to cause toxicity by interacting with mammalian cell membranes, the hemolytic activity of histone H5 versus rat erythrocytes was measured. In contrast to the other CAMPs mentioned above, rat RBCs incubated with increasing concentrations of histone H5 did not show significant hemolysis, even at H5 concentrations up to1 mg/mL (Table 4; P ≤ 0.05).

Due to their broad-spectrum of bactericidal activity and unique mechanism of action, CAMPs have attracted attention as potential therapeutic alternatives to antibiotics. However, a major barrier preventing the exploitation of natural peptides as therapeutics is the high cost for large scale manufacturing^20^. In fact, it has been estimated that it costs between $50-$400 to produce one gram (estimated average daily dose) of an antimicrobial peptide by solid-phase chemical synthesis^45^. Therefore, an effective strategy for reducing the manufacturing cost is the use of shorter peptides^20,21^. In this study, an *in silico* analysis was performed based on the most important characteristics of antimicrobial peptides (positive charge, hydrophobicity); six histone H5-derived peptide sequences composed of either 20 or 30 amino acids, with potential antimicrobial activity, were identified (full length of histone H5 is 190 a.a.). All of these sequences are located within amino acid positions 15–100, possibly representing the active antimicrobial domain(s) of histone H5 (Fig. 5). Interestingly, amino acid positions 20–109 represents the globular domain of histone H5 which folds into a helical bundle containing three α-helices, and possesses the two DNA-binding sites which are required for nucleosome organization^46,47^. The antimicrobial properties of the histone H5-inspired synthetic peptides were tested against *P. aeruginosa* and *L. monocytogenes*, which demonstrated that peptide H5(61–90) had the most potent activity (Table 6). Reasons for this superior antimicrobial activity could be due to the secondary structure that this peptide folds into upon contact with bacterial membranes and the high content of hydrophobic residues (46%) of this peptide, compared to the other H5-derived peptides. In fact, it has been shown that increasing the hydrophobicity of α-helical peptides to a certain degree is correlated with increased antimicrobial activity^48^. Once the most potent histone H5-derived peptide was identified following the initial screen for antimicrobial activity (H5(61–90) V1), the peptide was synthesized at >95% purity with chloride (H5(61–90) V2) or trifluoroacetate (H5(61–90) V3) counterions, and tested against *P. aeruginosa* and *L. monocytogenes*. Interestingly, both peptides synthesized at >95% purity demonstrated an unexpected decrease in antimicrobial activity against *L. monocytogenes* compared to the initial, less pure version of this peptide. However, even though *L. monocytogenes* displayed less susceptibility to H5(61–90) V2 and H5(61–90) V3, all three versions of the peptide H5(61–90) possessed similar MICs against *P. aeruginosa* (Table 7). Despite our identification of histone H5-derived peptides with the most potent predicted antimicrobial activity, the full length histone H5 protein inhibited *P. aeruginosa* and *L. monocytogenes* bacterial growth at significantly lower MICs than any of these peptides (P ≤ 0.05). For instance, histone H5 had MIC values of 0.2 ± 0 µM and 0.1 ± 0.1 µM against *L. monocytogenes* and *P. aeruginosa*, respectively; while the most potent H5-derived peptide, H5(61–90) V1, exhibited MIC values of 7.4 ± 2.6 µM (*L. monocytogenes*) and 5.1 ± 0 µM (*P. aeruginosa*). In some cases, it has been reported that shorter peptides display more potent antimicrobial activity than their parent peptide. For example, buforin I, a 39-amino acid CAMP isolated from the stomach of the Asian toad *Bufo bufo garagrizans*, is less active against Gram-positive bacteria, Gram-negative bacteria and fungi than the 21-amino acid derived CAMP buforin II^12^. In other examples, however, peptide derivatives display reduced antimicrobial activity than their parent peptides. For instance, arasin I, a 37-amino acid CAMP isolated from the spider crab has more potent antimicrobial activity against a broad range of microorganisms compared to the arasin I-derived peptides arasin I(1–20), arasin I(1–14) and arasin I(9–23)^49^. Correspondingly, compared to the histone H5-derived peptides tested in this study, further study with peptides of different lengths (shorter or longer) will be necessary to identify sequences with optimal antimicrobial activity.

The secondary structural components of peptides are important factors that influence their antimicrobial properties. For instance, as seen with buforin II and LL-37, an increase in α-helical content is associated with stronger antimicrobial activity^50^. The secondary structures of histone H5 and the three versions of histone H5-derived peptide H5(61–90) were analysed using circular dichroism (CD) spectroscopy and the majority of the peptides displayed an unordered random coil conformation (Table 8). This is not surprising, since many α-helical CAMPs have disordered secondary structures in aqueous solutions and fold into amphipathic α-helical conformations upon interaction with bacterial membranes^25,51^. For example, four cathelicidin PMAP-36-derived peptides displayed random coil conformations in 10 mM PBS (aqueous environment) and then folded into amphipathic α-helical conformations in the presence of trifluoroethanol (mimicking the hydrophobic environment of the bacterial membrane) or SDS (mimicking the negatively charged bacterial membrane)^21^. Similarly, LL-37 is converted from a random coil to an α-helix in the presence of Lipid A^52^. These examples demonstrate that CAMPs have the ability to fold into amphipathic α-helical conformations in membrane-mimetic environments, which we also observed for histone H5 and the three versions of peptide H5(61–90), the histone H5-derived peptide that displayed the most potent antimicrobial activity.

CAMPs have considerable advantages as alternative anti-infective compounds (rapid broad-spectrum antimicrobial activity and limited emergence of bacterial resistance), however, they also possess limitations for drug development. Examples include, potential toxicity of CAMPs and proteolytic degradation. In order to overcome these obstacles, introduction of D-form amino acids, acetylation or amidation of the terminal regions, and liposome encapsulation are methods that can improve the stability and reduce potential toxicity by CAMPs^45^. Consequently, in order to develop histone H5 and histone H5-derived peptides as candidates for therapeutic applications, these considerations must be investigated in future studies.

## Conclusion

In this study, histone H5 has been established, for the first time, as a protein with broad-spectrum antimicrobial activity which is highly effective against Gram-positive and Gram-negative planktonic bacterial pathogens, including resistant strains such as MRSA and VRE. Additionally, histone H5 significantly disrupted established biofilms of *L. monocytogenes* and *P. aeruginosa*. While other CAMPs such as LL-37, CAMA and paenibacterin have shown anti-biofilm properties, histone H5 is the first purified histone protein, to our knowledge, to demonstrate complete biofilm eradication activity. Furthermore, the MBC/MIC ratio and the SEM results demonstrate that histone H5 is bactericidal, and induces significant membrane damage leading to pronounced deformations, pore formation and leakage of cytoplasmic contents. This membrane-damaging activity, however, is limited to bacterial cells, since histone H5 was non-hemolytic towards mammalian erythrocytes. Considering these appealing characteristics, histone H5 is a bioinspiration for the development of novel histone H5-derived peptides. Overall, these findings demonstrate that histone H5 is a potent, broad-spectrum antimicrobial protein with novel potential for therapeutic applications in the clinical setting, as well as in food and agriculture industries, which are necessary as we advance towards a post-antibiotic era.

## Materials and Methods

### Bacterial strains and growth conditions

The bacterial strains tested in this study were Gram-positive bacteria (*Bacillus cereus* (ATCC 11778), methicillin-sensitive *Staphylococcus aureus* (ATCC 6538), methicillin-resistant *Staphylococcus aureus* (ATCC 29247), *Listeria monocytogenes* (ATCC 19112), *Enterococcus faecalis* (clinical isolate) and vancomycin-resistant *Enterococcus* (ATCC 51299)) and Gram-negative bacteria *Escherichia coli* K12 (ATCC 29425), *Escherichia coli* (O157:H7), *Pseudomonas aeruginosa* (ATCC 15442) and *Salmonella typhimurium* (ATCC 1535)). Bacterial colonies from glycerol stocks were plated on LB agar plates (BHI agar plates for *L. monocytogenes*) and incubated overnight at 37 °C. Single colonies from these plates were grown in 3 mL of LB broth (BHI broth for *L. monocytogenes*) overnight at 37 °C and shaken at 250 rpm. The inocula were diluted 1:50 in fresh LB broth (BHI broth for *L. monocytogenes*), incubated at 37 °C and shaken at 250 rpm until it displayed exponential growth (OD_600_ = 0.2). The bacterial suspension was pelleted at 3,000 × g, 4 °C for 10 minutes and washed with PBS (twice), and then adjusted to 10^5^–10^6^ CFUs/mL in PBS.

### Scanning electron microscopy (SEM)

The same protocols as described in the preparation of the bacterial suspension and the broth microdilution assay were followed to prepare the histone H5-treated bacteria and the untreated control cells for SEM. After a 3-hour incubation of the 1:1 (v/v) ratio of planktonic bacteria and histone H5 (or 1:1 (v/v) ratio of planktonic bacteria and sterile H_2_O for the untreated control cells), 100 µL of this solution was filtered directly through an Isopore™ polycarbonate membrane filter (0.2 μM pore size, EMD Millipore Co Cork, IRL) which was placed in a Swinny Stainless Steel 13 mm Filter Holder (EMD Millipore, MA, USA) connected to vacuum suction. The filter was then removed and placed onto a Kimwipe™ (Kimberly-Clark™ Professional Kimtech Science™) in a glass cell culture dish. The filters were fixed with 5% glutaraldehyde in 0.1 M sodium cacodylate, pH 7.5 (VWR, Radnor, PA, USA) overnight at 4 °C. The fixative was removed and the samples were dehydrated using sequential ethanol washes of 20, 40, 60, 80, 90, 95, and twice in 100% for 10 minutes each. Filters were then treated with 1:2 - hexamethyldisilizane (HMDS):100% ethanol, 2:1 - HMDS:100% ethanol and 100% HMDS (twice) for 10 min each (Sigma Aldrich, Oakville, ON, Canada). Filters were air dried overnight in the fume hood, sputter coated with gold to ~10–15 nm thickness and viewed using a Tescan Vega-II XMU VPSEM instrument at the Carleton University Nano Imaging Facility (Ottawa, Ontario, Canada).

### Determination of minimum biofilm eradication concentration (MBEC) of purified histone H5 versus bacterial biofilms

The preparation of the bacterial suspension was the same as described in the determination of the MIC and MBC, except that the bacterial suspension was adjusted to 10^5^ CFUs/ml in LB broth (BHI broth for *L. monocytogenes*), not PBS. Wells of an MBEC™ Assay Biofilm Inoculator (Innovotech, Edmonton, AB), which contains pegs projecting downwards from the lid that serve as a surface for biofilm formation, were inoculated with 150 µL of bacterial suspension. The plate was incubated for 24 hours at 37 °C (110 rpm) for *Pseudomonas aeruginosa* bacterial biofilms or for 72 hours at 22 °C (60 rpm) for *Listeria monocytogenes*. Following incubation, planktonic cells were rinsed away by switching the 96-peg lid to a sterile 96-well microplate with 200 μL of sterile PBS per well. Pegs with established biofilms were incubated with 200 μL of histone H5 (dissolved in sterile water, pH 7.4), in wells of another sterile 96-well microplate containing serially two-fold dilutions of H5. Solutions of gentamicin and kanamycin served as positive controls for *P. aeruginosa* and *L. monocytogenes*, respectively, whereas sterile water (pH 7.4) served as a negative control. The device was incubated for 2 hours at 37 °C, 110 rpm for *P. aeruginosa* and at 22 °C, 60 rpm for *L. monocytogenes*. The 96-peg lid was then rinsed twice as described above and placed on a sterile 96-well microplate with 200 μL of LB broth (*P. aeruginosa)* or BHI broth (*L. monocytogenes)* per well. The microplate was sonicated for 10 minutes (*P. aeruginosa*) or 30 minutes (*L. monocytogenes*) in order to dislodge the biofilms from the pegs, after which the peg-lid was replaced with a sterile, normal lid. This microplate was incubated in the EON microplate reader overnight at 37 °C (206 oscillations/min). The EON microplate spectrophotometer with Gen5 data analysis software (BioTek, Winooski, VT, USA) was used to monitor the growth of bacteria by measuring the optical density at 600 nm every 30 minutes for 24 hours (*P. aeruginosa*) or 48 hours (*L. monocytogenes*). The lowest histone H5 concentration without visible bacterial growth was designated as the minimum biofilm eradication concentration (MBEC). Additionally, a standard curve for the number of viable bacteria in the inoculum versus bacterial growth lag time was generated, since every microplate additionally contained wells with serially ten-fold diluted bacteria obtained from the uninhibited biofilm control (sterile H_2_O). Bacterial growth inhibition for each histone H5 concentration tested was determined from this standard curve.

### Bioinformatics analysis

Considering the general characteristics of antimicrobial peptides, a bioinformatics analysis was performed to identify histone H5-derived peptides with potential antimicrobial properties. The full length histone H5 protein sequence (total of 190 amino acid residues) was divided into sections of 20 amino acids (a.a) sequentially (example: section #1 would correspond to a.a. number 1 to 20 in the original H5 sequence and section #2 corresponds to a.a. number 21 to 40, etc.). Each sequence of 20 amino acids was then entered into the Antimicrobial Peptide Database (APD: http://aps.unmc.edu/AP/main.php) in order to obtain its properties related to antimicrobial activity, such as charge, hydrophobicity, the capacity to form alpha-helices, the number of hydrophobic residues on the same surface and the most similar AMP in the database. This original database is widely accepted and utilized in research and education^53^. The database includes >2900 AMPs, and categorizes peptides according to specific criteria: (i) natural peptides with a known sequence, (ii) antimicrobial activity and (iii) less than 200 amino acids in length^53^. In addition, the database also contains a frequently updated prediction interface which predicts if a sequence has the possibility to be an AMP based on parameters defined by all of the natural peptides entered in the database^53^. This analysis was also completed with sequential 20 amino acid sections starting at amino acid number 10 in the histone H5 sequence (example: section #1 would correspond to a.a. number 11 to 30 in the original H5 sequence and section #2 corresponds to a.a. number 31 to 50, etc.) and again with 30 amino acid sequences starting from a.a. number 1 and a.a. number 15 in the original histone H5 sequence. This *in silico* analysis identified six histone H5-derived peptides with predicted antimicrobial activity.

### Synthesis of the histone H5-derived peptides

Synthesis of the predicted histone H5-derived peptides was performed by Dr. Ajoy Basak and Mr. Chunyu Lu, University of Ottawa, using the solid-phase peptide synthesis method (SPPS)^54^ and the MultiPep automated peptide synthesizer (Intavis Bioinformatics Instruments). A total of 5 mg of lyophilized powder for each peptide was obtained, which was enough to perform a preliminary peptide screen to confirm antimicrobial activity. Additionally, samples of peptide H5(61–90) with a >95% purity were custom synthesized (GenScript), to obtain a 10–14 mg of lyophilized powder.

### Circular dichroism (CD) analysis

The protocol followed for circular dichroism spectrometry was based on previously described methods^55^. Circular dichroism (CD) measurements were performed using a Jasco-715 Spectropolarimeter (Japan Spectroscopic Co., Tokyo), in which the wavelengths scanned were 185 to 260 nm, using a scan speed of 100 nm/min, a bandwidth and data pitch of 1.0 nm, with a quartz cell (0.1 cm path length). Histone H5 and histone H5-derived peptides were dissolved in sterile ddH_2_O, pH 7.4, or 30 mM SDS. The CD spectrum of sterile ddH_2_O (buffer of the samples) was used as the blank. Each CD spectrum represents the average of two independent measurements, each in quintuplicate. The CD data was converted to molar ellipticity [θ], which was calculated using the following equation:

1 $$[{\rm{\theta }}]={\rm{CD}}\,{\rm{in}}\,\mathrm{mdeg}/({\rm{10}}\times {\rm{path}}\,{\rm{length}}\,{\rm{in}}\,\mathrm{cm}\times \mathrm{Cr})$$

where Cr = number of residues x molar concentration (mol/L).

In order to estimate secondary structure content, averaged CD spectra for each peptide were analysed using the CDSSTR software^56^. This program analyzes the molar ellipticity [θ] values at each wavelength and compares them with a library of known protein secondary structures (α-helix, β-sheet, turns, etc.) in order to estimate the percentages of the various secondary structural components of the tested peptide samples.

### Statistical analysis

Student’s T-test was used to determine the statistical significance of bacterial growth inhibition between the MIC and MBC values of histone H5, and to determine significant variations in bacterial biofilm growth and planktonic bacterial growth when calculating dose-dependent logarithmic inhibition. The Student’s T-test was also used to determine significant hemolytic activity of histone H5 compared to the PBS (no hemolysis) control and to compare the MIC values of the three versions of histone H5-derived peptide H5(61–90). In every case, a P value ≤ 0.05 was the threshold for statistical significance.

### Data Availability

All data generated or analysed during this study are included in this article (and its Supplementary Information files).

## Electronic supplementary material

Supplementary Information
