# Supplementary material for: Histone H5 is a potent Antimicrobial Agent and a template for novel Antimicrobial Peptides
Source: Sci Rep. 2018 Feb 5;8:2411. doi: 10.1038/s41598-018-20912-1 (PMC5799255; doi:10.1038/s41598-018-20912-1)
Supplement: Supplementary file 1 — Supplementary Information [file 41598_2018_20912_MOESM1_ESM.pdf]

Histone H5 is a potent Antimicrobial Agent and a template for novel Antimicrobial Peptides

Joelle Jodoin[1] and Maxwell T. Hincke[1, 2]*

Affiliations:

[1] Department of Cellular and Molecular Medicine, Faculty of Medicine, University of Ottawa, Ontario, Canada K1H 8M5.

[2] Department of Innovation in Medical Education, Faculty of Medicine, University of Ottawa, Ontario, Canada K1H 8M5.

***Corresponding Author:**

Maxwell T. Hincke, Department of Innovation in Medical Education, Faculty of Medicine, University of Ottawa, Ontario, Canada K1H 8M5.

Email: mhincke@uottawa.ca

Telephone: 613-562-5800 ext. 8193

Supplementary Information

Materials and Methods

Broth microdilution assay

The protocol for this step of the project was based on established methods[22]. Histone H5 or the peptides were dissolved in sterile water, pH 7.4, serially two-fold diluted and aliquoted in a 1:1 (v/v) ratio with bacteria. Kanamycin or ampicillin served as a positive control for inhibition, and sterile water (pH 7.4) as a negative control for inhibition. The tubes were then incubated for 3 hours at 37°C and shaken at 200 rpm. Next, 100 µL of each tube and 100µL of LB broth (BHI broth for *L. monocytogenes*) was plated in wells of a 96-well microplate. The microplate was incubated in the EON microplate reader overnight at 206 oscillations/min and at 37°C. During this period, the EON microplate spectrophotometer and Gen5 data analysis software (BioTek, Winooski, VT, USA) were used to monitor the growth of bacteria by measuring the optical density at 600 nm every 30 minutes for 18 hours. The lowest protein concentration without visible bacterial growth was designated as the minimum inhibitory concentration (MIC). Bacteria (10 µL) from the wells at MIC, MIC x 2 and MIC x 3 concentrations of H5 were plated on LB agar (BHI agar for *L. monocytogenes*) and incubated at 37°C for 18 hours. The lowest concentration of histone H5 without a single colony was designated as the minimum bactericidal concentration (MBC).

Hemolytic assay

This assay is based on a previously described protocol[22]. The potential for hemolytic activity was assessed using rat red blood cells (RBCs) which were obtained from the University of Ottawa Animal Care Facility and in accordance with the University of Ottawa Animal Care

Committee guidelines. Rat blood collected with EDTA (1 mL) was centrifuged at 1,000 x g for 10 minutes to pellet the RBCs. The serum was removed and the RBCs were washed with PBS, pH 7.4, and centrifuged at 1,000 x g for 10 minutes at room temperature (this was repeated three times). The RBCs were resuspended in PBS to a final volume of 1ml and then diluted 1:10 in PBS. The diluted RBCs were incubated in a 1:1 (v/v) ratio with different concentrations of histone H5 and incubated at 37°C with shaking at 200 rpm for 1 hour. Controls include PBS and PBS, 0.05% Triton-X, representing no hemolysis and 100% hemolysis, respectively. After the 1-hour incubation, the samples were centrifuged at 1,000 x g for 5 minutes and the absorbance of the supernatant was measured at 540 nm to monitor the release of hemoglobin. The percent hemolysis was calculated according to the following equation:

Hemolysis (%) = ((OD540 nm sample – OD540 nm no hemolysis)/(OD540 nm 100% hemolysis – OD540 nm no hemolysis)) x 100 (1)
